# Use of latent class analysis and patient reported outcome measures to identify distinct long COVID phenotypes: A longitudinal cohort study

**DOI:** 10.1371/journal.pone.0286588

**Published:** 2023-06-02

**Authors:** Alyson W. Wong, Karen C. Tran, Mawuena Binka, Naveed Z. Janjua, Hind Sbihi, James A. Russell, Christopher Carlsten, Adeera Levin, Christopher J. Ryerson

**Affiliations:** 1 Department of Medicine, University of British Columbia, Vancouver, Canada; 2 Centre for Heart Lung Innovation, St. Paul’s Hospital, University of British Columbia, Vancouver, Canada; 3 Division of General Internal Medicine, Department of Medicine, University of British Columbia, Vancouver, British Columbia, Canada; 4 Data and Analytic Services, BC Centre for Disease Control, Vancouver, British Columbia, Canada; 5 School of Population and Public Health, The University of British Columbia, Vancouver, British Columbia, Canada; The University of Mississippi Medical Center, UNITED STATES

## Abstract

**Objectives:**

We sought to 1) identify long COVID phenotypes based on patient reported outcome measures (PROMs) and 2) determine whether the phenotypes were associated with quality of life (QoL) and/or lung function.

**Methods:**

This was a longitudinal cohort study of hospitalized and non-hospitalized patients from March 2020 to January 2022 that was conducted across 4 Post-COVID Recovery Clinics in British Columbia, Canada. Latent class analysis was used to identify long COVID phenotypes using baseline PROMs (fatigue, dyspnea, cough, anxiety, depression, and post-traumatic stress disorder). We then explored the association between the phenotypes and QoL (using the EuroQoL 5 dimensions visual analogue scale [EQ5D VAS]) and lung function (using the diffusing capacity of the lung for carbon monoxide [DLCO]).

**Results:**

There were 1,344 patients enrolled in the study (mean age 51 ±15 years; 780 [58%] were females; 769 (57%) were of a non-White race). Three distinct long COVID phenotypes were identified: Class 1) fatigue and dyspnea, Class 2) anxiety and depression, and Class 3) fatigue, dyspnea, anxiety, and depression. Class 3 had a significantly lower EQ5D VAS at 3 (50±19) and 6 months (54 ± 22) compared to Classes 1 and 2 (p<0.001). The EQ5D VAS significantly improved between 3 and 6 months for Class 1 (median difference of 6.0 [95% CI, 4.0 to 8.0]) and Class 3 (median difference of 5.0 [95% CI, 0 to 8.5]). There were no differences in DLCO between the classes.

**Conclusions:**

There were 3 distinct long COVID phenotypes with different outcomes in QoL between 3 and 6 months after symptom onset. These phenotypes suggest that long COVID is a heterogeneous condition with distinct subpopulations who may have different outcomes and warrant tailored therapeutic approaches.

## Introduction

Protracted recovery after coronavirus disease 2019 (COVID-19) is common, with nearly 75% of patients reporting at least one persistent symptom 3 months after the acute illness, resulting in “long COVID” [1]. In a systematic analysis of post-COVID-19 symptoms, there were 45 studies with nearly 10,000 patients and 84 discrete symptoms [1]. The lack of widely accepted diagnostic criteria has hindered the ability to compare and aggregate data and characterize long COVID. There is also a crucial need to understand the biologic mechanisms of long COVID, identify those at risk of developing this condition, and determine its long-term outcomes.

A phenotype is the collection of observable traits that are influenced by a person’s genetics and their environment [2]. Given the heterogeneity in symptoms associated with long COVID, it is plausible that there are different underlying biologic mechanisms. The classification of disease phenotypes is important because this information can be used to predict prognosis, select patients for enrollment into clinical trials, personalize treatment, and provide the foundation for studies exploring pathobiology [2]. Studies investigating long COVID phenotypes have typically included symptoms identified from patient-completed questionnaires using a checklist format. The variability in symptoms included in studies and lack of standardized thresholds to categorize the presence of symptoms has led to heterogeneous phenotypes being identified and difficulties comparing findings between studies [3–5]. The use of standardized and validated tools to evaluate patient symptoms in long COVID is important, particularly since the diagnosis and monitoring of this condition is based on the presence of symptoms and their severity.

Patient reported outcome measures (PROMs) are standardized validated questionnaires completed by patients to provide information on their perceived functional well-being and health status [6]. The use of PROMs to determine long COVID phenotypes is particularly important as it ensures objective identification of symptoms and grading of their severity, analysis of symptoms longitudinally, and comparison with other diseases and healthy populations.

Another important aspect of establishing phenotypes is to ensure that they are associated with meaningful outcomes. Quality of life (QoL) is significantly reduced after COVID-19 [7]. The EuroQol 5-Dimensions (EQ5D) is a common QoL questionnaire comprised of a descriptive section (evaluates how mobility, self-care, usual activities, pain/discomfort and anxiety/depression are impacted) and a visual analogue scale (VAS) [8]. The EQ5D VAS allows the patient to rate their health on a scale of 0–100 with higher values representing better health. Lung function can also be impaired after COVID-19, with the diffusing capacity of the lung for carbon monoxide (DLCO) being the most affected pulmonary function measurement [9–12].

A challenge in studying the underlying biologic mechanisms in long COVID has been the tremendous number of reported symptoms and limited understanding of how best to connect clinical and translational research. Phenotypes can help filter which symptoms are critical to evaluating long COVID, show how symptoms may relate to one another, and in turn, inform discovery of underlying pathophysiologic pathways. The objectives of this study were therefore to 1) use a latent class analysis approach to identify and characterize long COVID phenotypes and 2) explore whether they are associated with quality of life (using the EQ5D VAS) and lung function (using the DLCO).

## Methods

### Study population

This is a longitudinal cohort of patients seen in one of the four Post-COVID-19 recovery clinics (PCRCs) located across British Columbia, Canada between March 2020 and January 2022. The PCRCs are comprised of a multidisciplinary team that includes physicians and allied health professionals (e.g., physiotherapists and nurses). Patients who were hospitalized with COVID-19 or treated as outpatients were included. Patients were eligible if they were over the age of 18 years, had persistent symptoms ≥12 weeks after the acute illness with SARS-CoV-2 infection confirmed by real-time reverse transcriptase–polymerase chain reaction (PCR), and had long COVID with complete baseline PROM data. Patients were considered to have long COVID if they had at least 1 abnormal PROM as outlined below. Ethics approval for this study was obtained from the Research Ethics Board at the University of British Columbia (H21-02660) and patients provided informed written consent.

### Measurements

Patients were assessed in the PCRC at 3 and 6 months after the onset of symptoms associated with their first PCR confirmed SARS-CoV-2 infection. Through chart reviews, we collected clinical data that included patient demographics and features of their acute COVID-19 illness (e.g., symptom onset date, hospitalization, and need for mechanical ventilation). Symptom data were collected using PROMs that were part of the questionnaires that patients completed at 3 and 6 months (abnormal scores based on previous literature are shown in parentheses): [13–18] Cough Visual Analogue Scale (≥30/100), University of California San Diego shortness of breath questionnaire (≥10/120), Fatigue Severity Scale (≥4/7), Generalized Anxiety Disorder 2-item (≥3/6), Patient Health Questionnaire-2 (≥3/6), Primary Care Post Traumatic Stress Disorder Screen for DSM-5 (≥3/5).

We explored outcomes using the EQ5D VAS and percent-predicted DLCO. The EQ5D VAS was collected at 3 and 6 months after symptom onset. Pulmonary function tests (PFTs), which include DLCO, were obtained if clinically indicated and were not part of the standardized longitudinal data that was collected. PFTs completed within 3 months of the baseline PROM questionnaire were used to analyze the relationship between phenotypes and DLCO.

### Statistical analysis

Latent class analysis (LCA) is a statistical method that uses responses to a set of indicators to identify unobserved groups of people that are similar to one another (“latent classes”) [19]. The benefit of using LCA compared to other classification techniques, such as cluster analysis, is that it permits a mathematical evaluation of how well a proposed LCA model represents the data.

We used LCA to identify groups of patients within our long COVID cohort based on similarities in their baseline PROM scores (normal versus abnormal). Multiple models were estimated by varying the number of classes. The analysis was conducted in a step-wise manner where classes were sequentially added until the model was overfit. The fit of each model was assessed using the Bayesian information criterion (BIC) and the Akaike information criterion (AIC), with lower values reflecting better fit [19]. There is no single parameter used to determine the optimal number of classes. Rather the combination of statistical fit indices, model interpretability and utility, parsimony, and clinical relevance of the classes is used to inform this decision.

After identifying the final model, phenotypes for each class were determined based on the PROMs that had the highest probability of being abnormal. Kruskal-Wallis and Chi-square tests were used to compare continuous and categorical variables, respectively, between the classes. A Kruskal-Wallis test was used to compare the EQ5D VAS between classes at 3 and 6 months after symptom onset. A Wilcoxon-test was used to determine if there was significant change in EQ5D between 3 and 6 months for each class. Linear regression was used to determine if there was an association between classes and DLCO at 3 months after adjusting for age, sex, and smoking history.

The change in EQ5D VAS over time was also evaluated using the minimum important difference (MID), which is the smallest change that is considered meaningful to a patient [20]. The MID for the EQ5D VAS in patients after COVID-19 is unknown, but ranges between 8–10 for patients with other respiratory conditions [21–23]. In our study, we used the more conservative MID threshold of 10 for the EQ5D VAS to avoid overestimating the proportion of patients who experienced a meaningful change. Statistical analyses were performed using R Version 4.0.3. A p-value < 0.05 was considered significant.

## Results

### Patient characteristics

There were 1,344 patients who met the study inclusion criteria. The cohort had a mean age of 51 years, was comprised of 58% females, and had a diverse patient population with 57% of people being of a non-White race (S1 Table). The majority of people did not require hospitalization during their acute illness (58%). The most common comorbidities were depression (38%), hypertension (29%), and asthma (19%). Fatigue and dyspnea were the most likely PROMs to be reported as abnormal. The median baseline EQ5D VAS was 60, which is below the age-matched population norm of 76 [24].

### Latent classes

Using latent class analysis, the fit statistics were determined for each model as the number of latent classes was sequentially increased (S2 Table). The final number of classes was 3 based on the model having the best fit (lower AIC and BIC values), being the most parsimonious (i.e., explained the data with the fewest classes), and having identified distinct groups of PROMs with clinically meaningful phenotypes.

### Long COVID phenotypes

**Fig 1:**
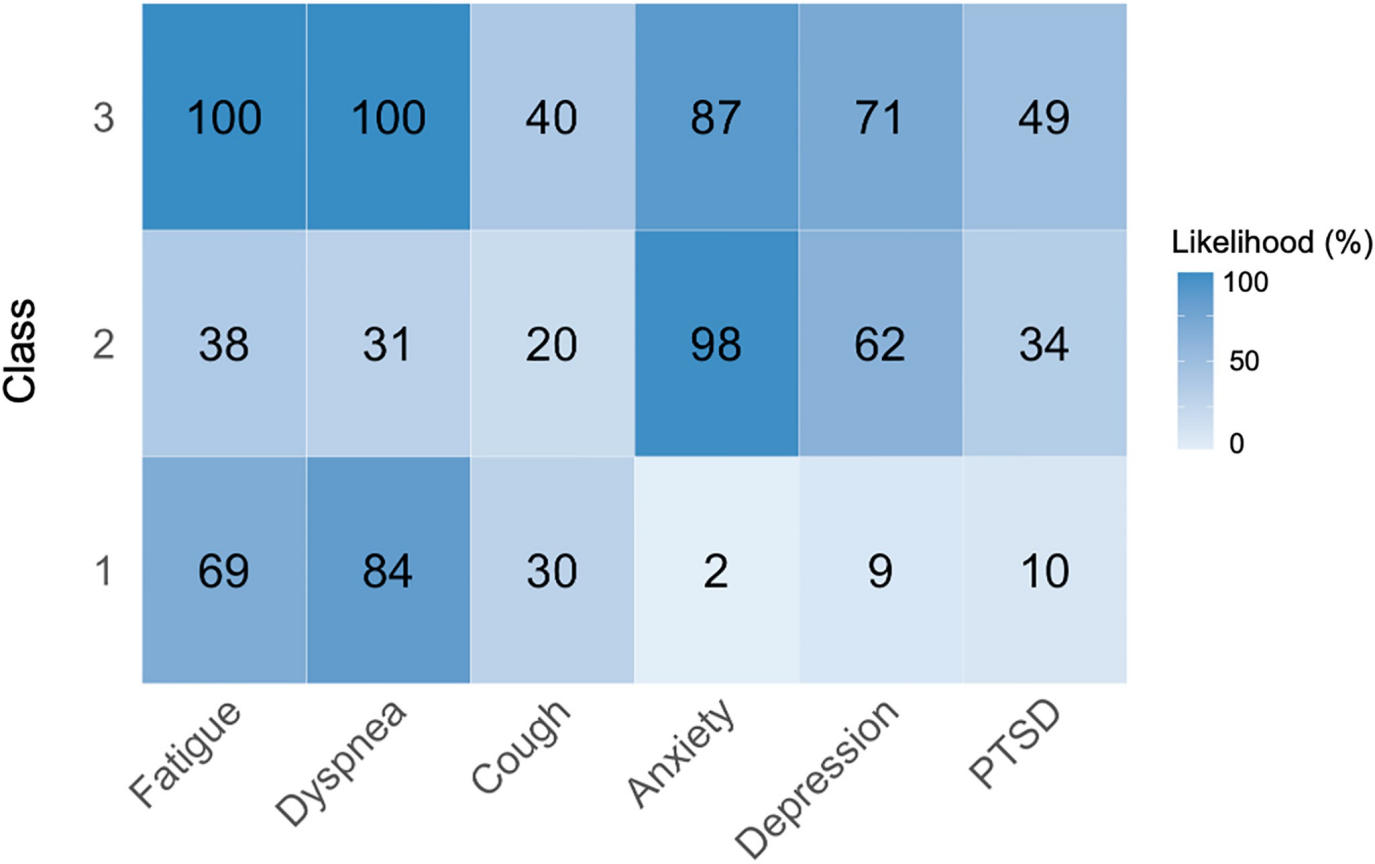
Likelihood of a PROM being abnormal for each class. Phenotypes for the classes are as follows: Class 1 = fatigue and dyspnea, Class 2 = anxiety and depression, Class 3 = fatigue, dyspnea, anxiety, and depression. Abbreviation: PTSD, post-traumatic stress disorder.

Phenotypes for each class were based on the likelihood of having an abnormal PROM: Class 1 was characterized mainly by patients who had fatigue and dyspnea, Class 2 by anxiety and depression, and Class 3 by fatigue, dyspnea, anxiety, and depression (Fig 1).

Compared to the other classes, patients in Class 3 (fatigue, dyspnea, anxiety, depression) were more likely to be female, be treated as outpatients during their acute illness, and have a higher likelihood of pre-existing asthma, chronic obstructive pulmonary disease (COPD), and depression (Table 1). Although classes had overlapping abnormal PROMs (e.g., Class 1 and 3 were both characterized by dyspnea), the severity of these abnormalities differed. In general, patients in Class 3 tended to have more severely abnormal PROMs compared to the other classes.

**Table 1:** Characteristics of patients within each latent class (n = 1,344).

			Class 1 (n = 769)	Class 2 (n = 93)	Class 3 (n = 482)	P value
Phenotype			Fatigue and dyspnea	Anxiety and depression	Fatigue, dyspnea, anxiety, depression	-
Age			53 ± 15	47 ± 15	49 ± 14	<0.001
Male sex, n (%)			349 (45)	36 (39)	179 (37)	0.013
BMI			29 ± 6	28 ± 7	29 ± 7	0.15
Hospitalized, n (%)			372 (48)	34 (37)	153 (32)	<0.001
ICU, n (%)			147 (19)	10 (11)	55 (11)	<0.001
Ever smoker, n (%)			202 (26)	26 (28)	146 (30)	0.32
Comorbidities, n (%)						
Coronary artery disease			90 (12)	5 (5)	58 (12)	0.16
Diabetes			147 (19)	16 (17)	73 (15)	0.20
Hypertension			226 (29)	25 (27)	142 (29)	0.87
Asthma			124 (16)	17 (18)	121 (25)	<0.001
COPD			44 (6)	3 (3)	53 (11)	<0.001
Malignancy			25 (3)	0 (0)	15 (3)	0.21
Depression			143 (19)	51 (55)	314 (65)	<0.001
PROMs	Score Range	Abnormal score				
Cough	0–100	≥ 30	0 (0–94)	0 (0–0)	0 (0–97)	<0.001
Dyspnea	0–120	≥ 10	25 (13–46)	6 (3–21)	44 (28–65)	<0.001
Fatigue	1–7	≥ 4	5 (3–6)	4 (3–5)	6 (6–7)	<0.001
Anxiety	0–6	≥ 3	1 (0–2)	4 (3–5)	4 (3–5)	<0.001
Depression	0–6	≥ 3	1 (0–2)	3 (2–4)	4 (2–5)	<0.001
PTSD	0–5	≥ 3	0 (0–1)	2 (0–3)	2 (1–4)	<0.001
EQ5D VAS (score range 0–100)						
3 months			64 ± 20	61 ± 22	50 ± 19	<0.001
6 months			67 ± 22	63 ± 22	54 ± 22	<0.001
Pulmonary function test (n = 249)						
FVC %-predicted			87 ± 19	96 ± 19	85 ± 21	0.16
DLCO %-predicted			81 ± 21	87 ± 16	78 ± 20	0.31

### Phenotypes and outcomes

#### QoL at 3 and 6 months

At 3 months after symptom onset, Class 3 (fatigue, dyspnea, anxiety, depression) had a significantly lower EQ5D VAS of 50 ± 19 compared to Class 1 (fatigue and dyspnea) EQ5D VAS of 64 ± 20 and Class 2 (anxiety and depression) EQ5D VAS of 61 ± 22 (both p<0.001). Similarly, at 6 months after symptom onset, Class 3 had a lower EQ5D VAS compared to the other classes. There was no significant difference in EQ5D VAS between Classes 1 and 2 at 3 months (64 ± 20 vs. 61 ± 22, p = 0.75) or 6 months (67 ± 22 vs. 63 ± 22, p = 0.66).

#### Change in QoL between 3 and 6 months

**Fig 2:**
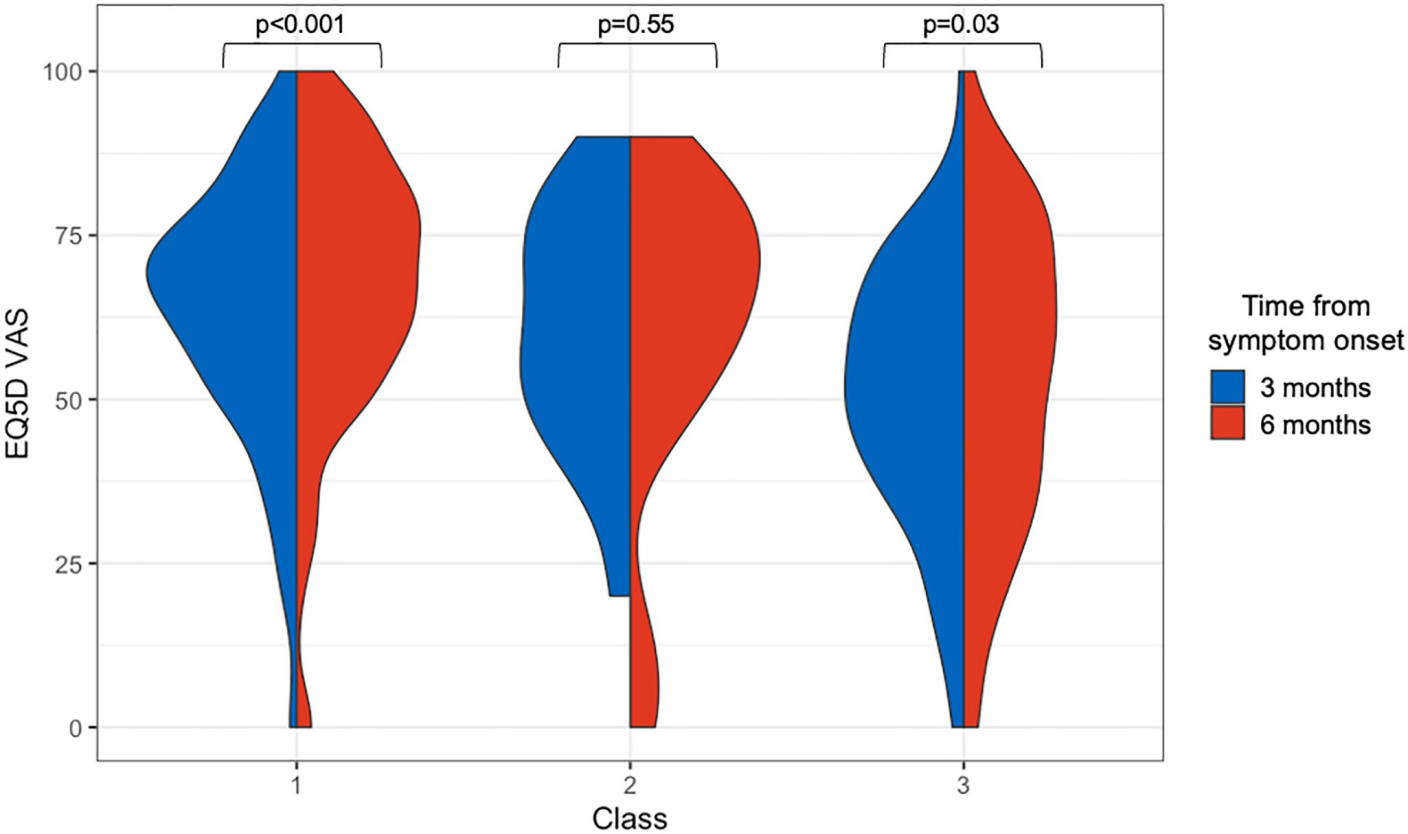
EQ5D VAS at 3 and 6 months after symptom onset based on latent classes. Class 3 had significantly lower EQ5D at 3 and 6 months compared to the other classes. Only patients in Classes 1 and 3 had a significant change in EQ5D VAS over this time. Class 1 = fatigue and dyspnea, Class 2 = anxiety and depression, Class 3 = fatigue, dyspnea, anxiety, and depression.

The change in EQ5D VAS between 3 and 6 months was ≥ 10 points (the MID for the EQ5D VAS) for 57% of the entire cohort. Thirty-seven percent of the cohort had a clinically meaningful improvement in quality of life, while 20% had a clinically meaningful decline between 3 and 6 months (S1 Fig). The change in EQ5D VAS over time increased for Class 1 (fatigue and dyspnea; median change 6 points [95% CI 4–8, p<0.001]) and Class 3 (fatigue, dyspnea, anxiety, depression; median change 5 points [95% CI 0–8.5, p = 0.03]) (Table 2 and Fig 2). Given Classes 1 and 3 were both characterized by fatigue and dyspnea, a post-hoc analysis was performed to evaluate whether the improvement in EQ5D could be explained by the changes in these symptoms. The fatigue and dyspnea scores did improve over time for these classes (S3 Table). However, there was weak correlation between the changes in dyspnea or fatigue scores and the change in EQ5D VAS (correlation coefficients -0.31 or -0.27 respectively for Class 1 and -0.25 or -0.17 respectively for Class 3).

**Table 2:** Change in EQ5D VAS between 3 and 6 months after symptom onset for each latent class. Class 1 = fatigue and dyspnea, Class 2 = anxiety and depression, and Class 3 = fatigue, dyspnea, anxiety, and depression.

Outcome	Class	Median difference	95% CI	P value
Change in EQ5D VAS over time	1	6.0	4.0 to 8.0	<0.001
	2	3.8	-18.5 to 11.5	0.55
	3	5.0	0 to 8.5	0.03

#### DLCO at 3 months

There were 212 patients who had pulmonary function testing within 3 months of the baseline PROM questionnaire. None of the classes were associated with DLCO in the unadjusted or adjusted analyses (Table 3).

**Table 3:** Association between latent classes and DLCO %-predicted (n = 212). Models were adjusted for age, sex, and smoking history. Class 1 = fatigue and dyspnea, Class 2 = anxiety and depression, Class 3 = fatigue, dyspnea, anxiety, and depression.

			Unadjusted analysis			Adjusted analysis		
Outcome	Reference	Primary predictor	Coefficient	95%CI	P value	Coefficient	95%CI	P value
DLCO %-predicted at baseline	Class 1	Class 2	5.7	-6.8 to 18.2	0.37	0.02	-10.9 to 11.0	0.99
	Class 1	Class 3	-5.0	-11.2 to 1.2	012	-2.5	-7.9 to 2.9	0.36
	Class 2	Class 3	-10.7	-23.6 to 2.3	0.11	-2.5	-14.0 to 8.5	0.66

## Discussion

A major challenge faced by patients with long COVID has been the lack of objective diagnostic tests or biomarkers, variable patterns of presentation, uncertain pathophysiology, and heterogeneous outcomes, making it difficult to counsel patients on the anticipated recovery. In this study, we demonstrate that certain symptoms occur more frequently together resulting in discrete long COVID groups that have different outcomes. Specifically, we identified 3 latent classes with distinct phenotypes based on abnormal PROMs: Class 1) fatigue and dyspnea, Class 2) anxiety and depression, and Class 3) fatigue, dyspnea, anxiety, and depression. We also identified differences in outcomes among these phenotypes (i.e., QoL at 3 and 6 months and the rate of change in QoL during this time period).

There were several interesting differences between the phenotypes. Classes 2 and 3, which were both characterized by anxiety and depression, had a higher proportion of people treated as outpatients during their acute COVID-19 illness. The higher prevalence of anxiety and depression among these classes is likely multifactorial including the presence of pre-existing mental health conditions and patient expectations of recovery. Among those who experience mild disease and are treated as outpatients, there may be a discordance between their expected and actual recovery. As a result, the presence of persistent symptoms months after mild illness may impact mental health outcomes and overall quality of life.

There was a higher proportion of pre-existing pulmonary diseases (asthma and COPD) in Class 3 (fatigue, dyspnea, anxiety, and depression). This finding is consistent with previous research evaluating the relationship between psychosocial outcomes and respiratory conditions [25]. In one study, the prevalence of anxiety (28%) and depression (19%) among patients with COPD was higher than age- and sex-matched healthy subjects (6% and 4% respectively), regardless of COPD severity [25]. In particular, females had a higher prevalence of both anxiety and depression (26%) compared to males (7%) and reported worse dyspnea and symptom-related QoL [25].

The difference in inspiratory muscle strength has been proposed as a pathophysiologic mechanism for increased perception of dyspnea in women compared to men [26]. One clinical trial randomized women with mild-to-moderate asthma to receive inspiratory muscle training (until their maximum inspiratory pressure was equal to the male subjects) or sham training [26]. Initially, the female subjects had a higher perception of dyspnea compared to males; however, this difference disappeared for the female subjects who completed the training. The relationship between inspiratory muscle strength and dyspnea in patients with long COVID warrants further exploration. Interventions used to treat dyspnea in respiratory conditions could potentially benefit those with long COVID, although prospective clinical trials are required to better address these questions.

QoL improved for Classes 1 and 3 (both characterized by fatigue and dyspnea) but did not improve for Class 2 (anxiety and depression). There was a significant decrease in fatigue and dyspnea scores for both classes, however the improvement in these PROMs were weakly correlated with the change in EQ5D VAS and may not fully explain the improvements in QoL. Furthermore, there was no association between classes characterized by dyspnea and DLCO. These findings suggest that dyspnea in long COVID may be due to causes outside of pulmonary vascular abnormalities. Research into the underlying mechanisms and treatment of fatigue and dyspnea remain critical areas to prioritize.

Long COVID phenotypes have been identified in other studies. Frontera *et al*. describe 3 distinct phenotypes among hospitalized patients 12 months after positive SARS-CoV-2 testing (Cluster 1: few symptoms, Cluster 2: many symptoms with high frequency of anxiety and depression, Cluster 3: shortness of breath, headache, and cognitive symptoms) [3]. Another study similarly found clusters with varying severities of mental and physical health impairments [27]. The majority of these studies have evaluated patients who were hospitalized with COVID-19. Our study included both hospitalized and non-hospitalized patients and showed that phenotypes characterized by anxiety and depression were predominantly comprised of those with mild COVID-19 illness and treated in the outpatient setting. Given over 90% of patients with COVID-19 are not hospitalized [28], identifying long COVID phenotypes among these patients and determining whether there are differing outcomes and responses to treatment will be critical to help patients in their recovery and effectively allocate healthcare resources.

The identification of phenotypes was limited to the PROMs included in our analysis. Although we used a limited set of PROMs, these reflect some of the most common symptoms that persist after COVID-19 [1, 11, 29]. Other studies have typically included symptoms based on checklists that patients completed. A strength of this study is the use of PROMs which are validated tools that offer more granular data than a patient selecting ‘yes’ or ‘no’ to the presence of a symptom. The PROMs used in this study had a numerical score and validated thresholds had to be met in order for a patient to be classified as having a symptom. We also used data that were systematically collected from a large, real-world cohort of patients. The combination of these features increases the objectivity and generalizability of the identified phenotypes. Our findings require validation and, ideally, we would demonstrate reproducible results in an external cohort; however, different tools are being used to collect symptom data around the world. This heterogeneity in data collection highlights a major barrier to studying long COVID. Lastly, patients who were treated as outpatients for COVID-19 were referred to the PCRC because they had severe enough symptoms to seek medical attention and may not represent all patients who had mild COVID-19. Although this contributes to referral bias, the patients in our cohort reflect those who are most likely to require and benefit from intervention.

## Conclusions

Long COVID phenotypes were identified using patient reported outcome measures among a large, real-world cohort of hospitalized and non-hospitalized patients. These phenotypes suggest that long COVID is a heterogeneous condition with distinct subpopulations who may have different outcomes and warrant tailored therapeutic approaches.

## Supporting information

S1 Table Baseline characteristics for the entire cohort (n = 1,344). Values represent mean ± standard deviation, number (percent), or median (interquartile range). Other races include patients who declined to answer or selected the option for ‘Other race.’ EQ5D score ≥ 3 refers to patients who report at least moderate problems in the quality of life domain.
(PDF)

S2 Table Comparison of latent class models. The final model selected (Model B) was based on model fit (AIC and BIC), distribution of patients among the classes, the fewest number of classes being able to explain the data (i.e., parsimony), and identification of clinically relevant phenotypes. Abbreviation: AIC, Akaike information criterion; BIC, Bayesian information criterion; PTSD, post-traumatic stress disorder.
(PDF)

S3 Table Change in fatigue and dyspnea between 3 and 6 months after symptom onset for each latent class. Class 1 = fatigue and dyspnea, Class 2 = anxiety and depression, Class 3 = fatigue, dyspnea, anxiety, and depression. Abbreviation: UCSD, University of California San Diego Shortness of Breath Questionnaire.
(PDF)

S1 Fig Change in EQ5D VAS between 3 and 6 months (n = 734). There were 416 patients (57%) whose EQ5D VAS changed by at least 10 points, with 37% reporting a clinically meaningful improvement (green) and 20% reporting a clinically meaningful decline (red).
(PDF)

## Abbreviations

COPD: Chronic obstructive pulmonary disease
COVID-19: Coronavirus disease 2019
DLCO: Diffusing capacity of the lung for carbon monoxide
EQ-5D: EuroQoL 5 dimensions
GDP: Gross domestic product
LCA: Latent class analysis
MID: Minimum important difference
PROM: Patient reported outcome measure
QoL: Quality of life
VAS: Visual analogue scale
