# Supplementary material for: Use of latent class analysis and patient reported outcome measures to identify distinct long COVID phenotypes: A longitudinal cohort study
Source: PLoS One. 2023 Jun 2;18(6):e0286588. doi: 10.1371/journal.pone.0286588 (PMC10237387; doi:10.1371/journal.pone.0286588)
Supplement: S1 Table — Values represent mean ± standard deviation, number (percent), or median (interquartile range). Other races include patients who declined to answer or selected the option for ‘Other race.’ EQ5D score ≥ 3 refers to patients who report at least moderate problems in the quality of life domain. (PDF) [file pone.0286588.s001.pdf]

SUPPLEMENT

Table S1. Baseline characteristics for the entire cohort (n=1,344). Values represent mean ± standard deviation, number (percent), or median (interquartile range). Other races include patients who declined to answer or selected the option for 'Other race.' EQ5D score ≥ 3 refers to patients who report at least moderate problems in the quality of life domain.

Characteristics	Values
Age, years	51 ± 15
Male sex	564 (42)
Smoking pack-years	4 (1-16)
Race	
White	575 (43)
Asian	455 (34)
South Asian	212 (47)
East Asian	109 (24)
Southeast Asian	104 (23)
Other	30 (6)
Indigenous	51 (4)
Black	11 (1)
Latin American	40 (3)
Other	212 (15)
Employed	933 (69)
Acute COVID-19 Illness	
Hospitalized	559 (42)
ICU	212 (34)
Comorbidities, n (%)	
Coronary artery disease	153 (11)
Diabetes	236 (18)
Hypertension	393 (29)
Asthma	262 (19)
COPD	100 (7)
Malignancy	40 (3)
Depression	508 (38)
Abnormal patient reported outcomes, n (%)	
Cough	441 (33)
Dyspnea	1154 (86)
Fatigue	1048 (78)
Anxiety	529 (39)
Depression	468 (35)
PTSD	342 (25)
EQ5D score ≥ 3, n (%)	
Mobility	453 (34)
Self-care	139 (10)
Usual activities	718 (53)
Pain or discomfort	682 (51)
Anxiety or depression	610 (45)
EQ5D VAS	60 (50-75)
