# Supplementary material for: Use of latent class analysis and patient reported outcome measures to identify distinct long COVID phenotypes: A longitudinal cohort study
Source: PLoS One. 2023 Jun 2;18(6):e0286588. doi: 10.1371/journal.pone.0286588 (PMC10237387; doi:10.1371/journal.pone.0286588)
Supplement: S2 Table — The final model selected (Model B) was based on model fit (AIC and BIC), distribution of patients among the classes, the fewest number of classes being able to explain the data (i.e., parsimony), and identification of clinically relevant phenotypes. Abbreviation: AIC, Akaike information criterion; BIC, Bayesian information criterion; PTSD, post-traumatic stress disorder. (PDF) [file pone.0286588.s002.pdf]

Table S2. Comparison of latent class models. The final model selected (Model B) was based on model fit (AIC and BIC), distribution of patients among the classes, the fewest number of classes being able to explain the data (i.e., parsimony), and identification of clinically relevant phenotypes. Abbreviation: AIC, Akaike information criterion; BIC, Bayesian information criterion; PTSD, post-traumatic stress disorder.

Model	AIC	BIC	Class	% of cohort	Likelihood that the PROM will be abnormal (%)					
					Cough	Dyspnea	Fatigue	Anxiety	Depression	PTSD
A	8718	8786	1	61	32	82	70	12	10	9
			2	39	34	92	91	83	75	51
B	8673	8777	1	57	30	84	69	2	9	10
			2	7	20	31	38	98	62	34
			3	36	40	100	100	87	71	49
C	8665	8805	1	52	35	89	67	7	8	7
			2	35	38	100	98	78	71	52
			3	3	0	0	100	0	0	14
			4	10	18	48	55	85	61	29
D	8841	8818	1	10	0	100	0	1	3	4
			2	24	0	76	100	2	6	10
			3	18	100	84	71	11	7	5
			4	35	37	100	99	77	70	51
			5	12	15	55	53	78	55	30
