# Supplementary material for: Use of latent class analysis and patient reported outcome measures to identify distinct long COVID phenotypes: A longitudinal cohort study
Source: PLoS One. 2023 Jun 2;18(6):e0286588. doi: 10.1371/journal.pone.0286588 (PMC10237387; doi:10.1371/journal.pone.0286588)
Supplement: S3 Table — Class 1 = fatigue and dyspnea, Class 2 = anxiety and depression, Class 3 = fatigue, dyspnea, anxiety, and depression. Abbreviation: UCSD, University of California San Diego Shortness of Breath Questionnaire. (PDF) [file pone.0286588.s003.pdf]

Table S3. Change in fatigue and dyspnea between 3 and 6 months after symptom onset for each latent class. Class 1 = fatigue and dyspnea, Class 2 = anxiety and depression, Class 3 = fatigue, dyspnea, anxiety, and depression. Abbreviation: UCSD, University of California San Diego Shortness of Breath Questionnaire.

Outcome	Class	Median difference	95% CI	P value
Change in fatigue severity score over time	1	-0.4	-0.5 to -0.2	<0.001
	2	0.2	-0.4 to 0.9	0.38
	3	-0.4	-0.6 to -0.2	<0.001
Change in UCSD over time	1	-6.5	-8.0 to -4.5	<0.001
	2	0.5	-4.5 to 5.5	0.85
	3	-5.0	-8.0 to -2.5	<0.001
