# Supplementary material for: Use of latent class analysis and patient reported outcome measures to identify distinct long COVID phenotypes: A longitudinal cohort study
Source: PLoS One. 2023 Jun 2;18(6):e0286588. doi: 10.1371/journal.pone.0286588 (PMC10237387; doi:10.1371/journal.pone.0286588)

Figure S1. Change in EQ5D VAS between 3 and 6 months (n=734). There were 416 patients (57%) whose EQ5D VAS changed by at least 10 points, with 37% reporting a clinically meaningful improvement (green) and 20% reporting a clinically meaningful decline (red).

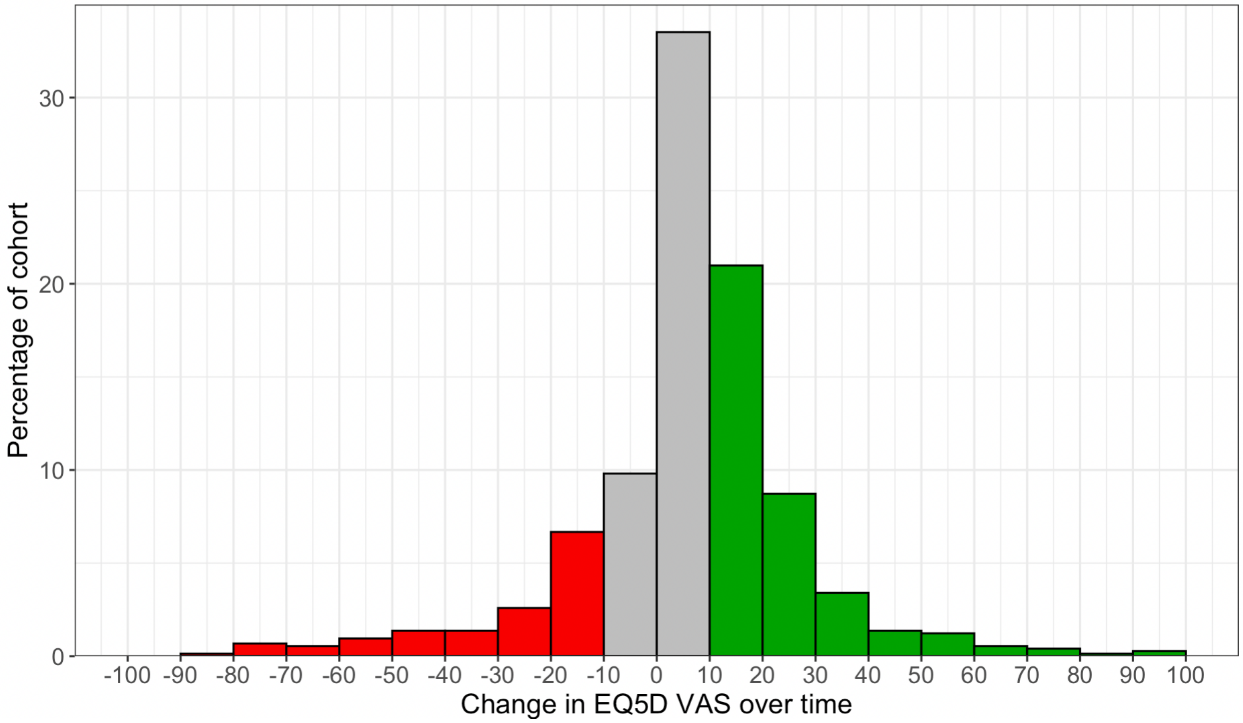

Supplement: S1 Fig — There were 416 patients (57%) whose EQ5D VAS changed by at least 10 points, with 37% reporting a clinically meaningful improvement (green) and 20% reporting a clinically meaningful decline (red). (PDF) [file pone.0286588.s004.pdf]
